# The role of N6-methyladenosine modification in tumor angiogenesis

**DOI:** 10.3389/fonc.2024.1467850

**Published:** 2024-12-03

**Authors:** Lifei Qin, Xinya Zeng, Xinze Qiu, Xingmei Chen, Shiquan Liu

**Affiliations:** Department of Gastroenterology, The Second Affiliated Hospital of Guangxi Medical University, Nanning, Guangxi, China

**Keywords:** N6-methyladenosine, writer, eraser, reader, tumor angiogenesis, tumor therapy

## Abstract

Tumor angiogenesis is a characteristics of malignant cancer progression that facilitates cancer cell growth, diffusion and metastasis, and has an indispensable role in cancer development. N6-methyladenosine (m6A) is among the most prevalent internal modifications in eukaryotic RNAs, and has considerable influence on RNA metabolism, including its transcription, splicing, localization, translation, recognition, and degradation. The m6A modification is generated by m6A methyltransferases (“writers”), removed by m6A demethylases (“erasers”), and recognized by m6A-binding proteins (“readers”). There is accumulating evidence that abnormal m6A modification is involved in the pathogenesis of multiple diseases, including cancers, and promotes cancer occurrence, development, and progression through its considerable impact on oncoprotein expression. Furthermore, increasing studies have demonstrated that m6A modification can influence angiogenesis in cancers through multiple pathways to regulate malignant processes. In this review, we elaborate the role of m6A modification in tumor angiogenesis-related molecules and pathways in detail, providing insights into the interactions between m6A and tumor angiogenesis. Moreover, we describe how targeting m6A modification in combination with anti-angiogenesis drugs is expected to be a promising anti-tumor treatment strategy, with potential value for addressing the challenge of drug resistance.

## 1 Introduction

Tumor cell proliferation relies on provision of sufficient oxygen and nutrients to meet the metabolic needs of the tissue via blood vessels; however, when tumors grow beyond a certain extent, existing blood vessels are insufficient to satisfy the demands of the tumor, and new blood vessel formation is required (1). During tumor angiogenesis, the “angiogenesis switch” is activated due to the imbalance of pro- and anti-angiogenic factors in the tumor microenvironment, resulting in formation of an abnormal tumor vascular system (2). Cancer vascular networks are also integral to the metastasis and spread of cancer tissue to distant organs.

In recent years, there has been considerable research interest in N6-methyladenosine (m6A) RNA methylation, which is an internal RNA modification occurring extensively in mammalian eukaryotic cells as an epigenetic gene expression regulatory mechanism (3). The m6A modification is most frequent found in highly-conserved RRACH (R=G/A, H=A/C/U) consensus sequences, and is predominantly enriched in 3’-untranslated terminal regions (3’-UTRs), close to termination codons, and in internal exons (4, 5). Further, m6A modification is a dynamic and reversible process that methylated at the N-6 site of adenosine of RNA molecule, not only presenting in messenger RNAs (mRNAs), but also in non-coding RNAs (ncRNAs), such as long ncRNAs (lncRNAs), microRNAs (miRNAs), and circular RNAs (circRNAs) (3, 6). The basic processes involved in m6A methylation are regulated by interactions among three factors: “writers” (m6A methyltransferases), “erasers” (m6A demethylases), and “readers” (RNA-binding proteins) (7), where m6A methyltransferases catalyze methylation modifications, m6A demethylases are responsible for removal of methylation modifications, and RNA-binding proteins are mainly responsible for recognizing and binding to specific m6A binding sites (8). Through its roles in processes including RNA transcription, splicing, localization, translation, recognition, and degradation, m6A methylation modification participates widely in the regulation of target RNA expression, which contributes to tumorigenesis and tumor progression (9). Research into m6A modification in the context of tumor angiogenesis has gradually increased and numerous molecular mechanisms related to tumor angiogenesis regulation by m6A have been preliminarily validated.

In this review, we provide a brief introduction to the biological processes of m6A modification and tumor angiogenesis. Then, we summarize studies on the mechanisms by which m6A modification impacts the development of diverse tumors through regulation of angiogenesis. Finally, we discuss the potential clinical application value of targeting m6A modification combined with drugs inhibiting angiogenesis in the treatment of cancer.

## 2 Regulators of m6A modification

As an epigenetic posttranscriptional regulatory mechanism and an emerging research frontier, m6A modification is the most prevalent and abundant internal chemical modification occurring in mammalian mRNAs and ncRNAs (10–13). m6A methylation was first discovered in 1974, and its study has increased considerably in recent years, owing to improvements in detection methods (3). m6A modification is involved in the regulation of almost all RNA metabolism processes, and influences various biological functions (8, 9, 14), including RNA transcription (15, 16), splicing (17–19), subcellular location (20, 21), translation (22, 23), stability (24–26) and binding capacity (27), thus affecting RNA expression and functions. In mammalian RNA, m6A accounts for approximately 0.1%–0.4% of adenylate residues, representing an average of 3-5 m6A-modified sites per transcript (5, 11, 28). m6A modification is defined as methylation of the sixth N atom of RNA adenylate (A) (3). With the development and application of high-throughput sequencing technologies, abundant studies have reported that m6A sites are preferentially enriched in 3’-UTRs, near termination codons, and in internal exons, at the highly-conserved consensus motif, RRACH (4, 5, 26, 29). In recent years, m6A modification sites have also been discovered 5’-UTRs, and play critical roles in commencement of cap-independent translation (30, 31).

**Figure 1:**
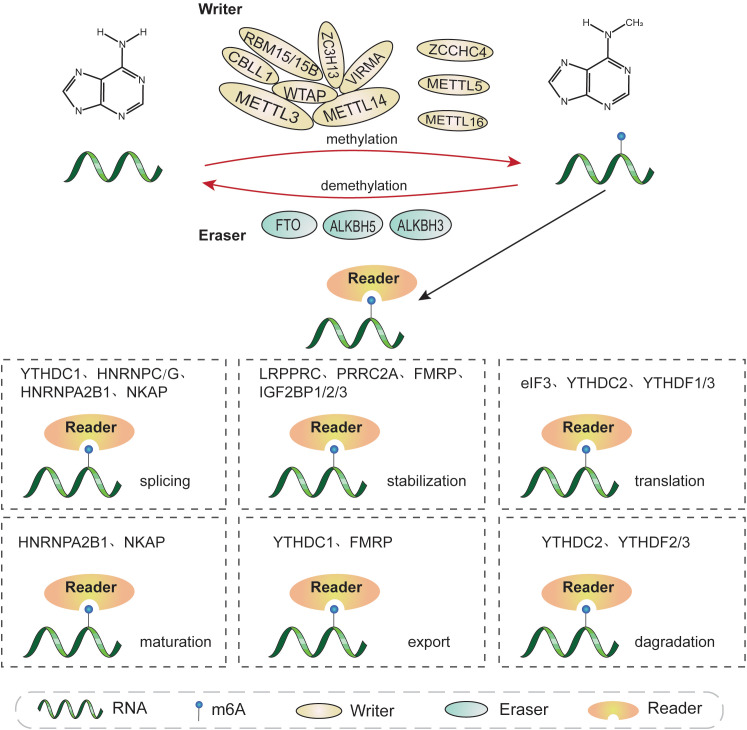
The molecular mechanisms involved in m6A methylation. m6A modification is a dynamic and reversible process conducted by m6A “writers”, “erasers”, and “readers”. m6A “writers” (METTL3, METTL14, WTAP, RBM15/15B, VIRMA, ZC3H13, CBLL1, ZCCHC4, METTL5, and METTL16) catalyze RNA methylation. m6A modification can be removed by m6A “erasers”, including FTO, ALKBH5, and ALKBH3. m6A “readers” recognize and combine with m6A sites on targeted RNA to affect its fate, and primarily include YTHDC1/2, YTHDF1/2/3, IGF2BP1/2/3, HNRNPA2B1, HNRNPC/G, eIF3, FMRP, PRRC2A, LRPPRC, and NKPA. m6A modifications are involved in almost all RNA metabolic processes, including transcription, splicing, translation, degradation, and nuclear export, among others.

Similar to DNA methylation and histone modifications, m6A RNA modification is dynamic and reversible, and is mediated by three categories of enzymes: methyltransferases, also referred to as “writers”, which catalyze methylation; demethylases, or “erasers”, which removed the modification; and RNA binding-proteins, known as “readers”, which recognize and bind to m6A sites (8, 32, 33). The functions of these proteins separately ensure the expression of RNAs (**Figure 1** **;** **Table 1** **).**

**Table 1:** The function of m6A methylation enzymes/regulators in RNA metabolism.

Type	Regulators	Full name	Function	Refs
Writers	METTL3	Methyltransferase-like 3	catalyzes m6A modification	(38)
	METTL14	Methyltransferase-like 14	intensifies METTL3 catalytic activity and combines with target RNAs	(39)
	WTAP	Wilms Tumor 1 associated protein	promotes METTL3-METTL14 heterodimer co-locates in nuclear speckles	(43)
	RBM15	RNA-binding motif protein 15	recruits of m6A complex to specific RNA-binding sites	(45)
	RBM15B	RNA-binding motif protein 15B	recruits of m6A complex to specific RNA-binding sites	(46)
	VIRMA(KIAA1429)	Vir-like m6A methyltransferase-associated	recruits the methyltransferase core complex to specific RNA sites and associates with cleavage polyadenylation specificity factors CPSF5 and CPSF6.	(48)
	ZC3H13	Zinc finger Cys-Cys-Cys-His (CCCH)-type containing 13	retains the MTC in nuclear speckles to enhance m6A modification	(49)
	CBLL1(Hakai)	Cbl Protooncogene-like 1	maintains the stability of m6A-METTL Associated Complex (MACOM)	(50)
	ZCCHC4	Zinc finger Cys-Cys-Cys-His (CCHC)-type containing 4	catalyzes 28S rRNA m6A modification	(51)
	METTL5	Methyltransferase-like 5	catalyzes 18S rRNA m6A modification	(53)
	METTL16	Methyltransferase-like 16	promote RNA translation in the cytoplasm and m6A deposition in the nucleus	(56)
Erasers	FTO	Fat mass and obesity-associated protein	removes m6A modification	(60)
	ALKBH5	AlkB homologue 5	removes m6A modification	(61)
	ALKBH3	AlkB homologue 3	removes m6A modification	(63)
Readers	YTHDC1	YT521-B homology (YTH) domain-containing 1	promotes pre-RNA splicing and RNA nuclear export	(71)
	YTHDC2	YT521-B homology (YTH) domain-containing 2	improves the translation efficiency and decreases the stability and abundance of target RNAs	(75)
	YTHDF1	YTH domain family protein 1	promotes mRNA translation initiation	(23)
	YTHDF2	YTH domain family protein 2	promotes mRNA degradation	(67)
	YTHDF3	YTH domain family protein 3	cooperates with YTHDF1 to promote mRNA translation or cooperates with YTHDF2 to promote mRNA degradation	(70)
	IGF2BP1	Insulin-like factor-2 mRNA-binding protein 1	promotes the stability and translation efficiency of target mRNA	(27)
	IGF2BP2	Insulin-like factor-2 mRNA-binding protein 2	promotes the stability and translation efficiency of target mRNA	(27)
	IGF2BP3	Insulin-like factor-2 mRNA-binding protein 3	promotes the stability and translation efficiency of target mRNA	(27)
	HNRNPA2B1	Heterogeneous nuclear ribonucleoprotein A2/B1	promotes mRNA splicing and miRNA maturation	(20)
	HNRNPC	Heterogeneous nuclear ribonucleoprotein C	mediates mRNA abundance and splicing	(18)
	HNRNPG	Heterogeneous nuclear ribonucleoprotein G	mediates mRNA abundance and splicing	(78)
	eIF3	Eukaryotic translation initiation factor 3	enhances mRNA translation	(31)
	FMRP	Fragile X mental retardation protein	modulates m6A-dependent mRNAs nuclear export and stability	(80)
	PRRC2A	Proline rich coiled-coil 2A	modulates target mRNA stability	(82)
	LRPPRC	Leucine-rich pentatricopeptide repeat-containing	heightens target PD-L1 mRNA stability	(85)
	NKAP	NF-κB-associated protein	facilitates target mRNA splicing and maturation	(84)
Others	H3K36me3	Histone H3 trimethylation at Lys36	promote m6A deposition in mammals	(87)
	H3K36me2	Histone H3 dimethylation at lysine 36	promote m6A deposition in the Arabidopsis genome	(88)
	H1	Histone H1	facilitate m6A deposition	(89)
	RNAPII	RNA polymerase II	suppress m6A deposition	(90)
	TARBP2	Trans-activation response (TAR) RNA-binding protein 2	promote m6A deposition	(91)
	EJCs	Exon junction complexes	suppress m6A deposition	(92)

### 2.1 m6A “writers”

The core components of the methyltransferase complex (MTC) are methyltransferase-like 3 (METTL3), methyltransferase-like 14 (METTL14), and Wilms tumor 1 associated protein (WTAP) (34, 35). METTL3 is a highly conserved S-adenosyl methionine (SAM)-binding protein identified by Joseph et al. in 1997 (36), and the most significant component subunit of the MTC, able to catalyze the transfer of methyl groups in SAM to adenine bases in RNA (37). As a pseudo-methyltransferase, METTL14 exhibits no catalytic activity; however, it plays an essential role in allosteric activation of METTL3, to intensify its catalytic function and is also responsible for combining with target RNAs by recognizing the specific RRACH consensus sequence (38, 39). A 1:1 ratio of METTL3 and METTL14 come together to form a stable heterodimer and colocalize in nuclear speckles (40). A recent study found that METTL3 is vital for promoting METTL14 stabilization by protecting its ubiquitinated sites from STIP1 homology and U-box-containing protein 1 (STUB1)-induced ubiquitination degradation to maintain m6A homeostasis (41). WTAP, a partner of the Wilms tumor 1 (WT1) protein, can promote recruitment of the METTL3-METTL14 heterodimer and ensure colocalization of the complex in nuclear speckles to mediate m6A modification (42). WTAP is a bridging protein that occurs in two different complexes: the METTL3-METTL14-WTAP complex, also referred to as the m6A–METTL complex (MAC), which has a primarily catalytic role; and the RBM15/ZC3H13/WTAP/VIRMA/Hakai complex, known as the m6A-METTL-associated complex (MACOM), which mainly exerts a regulatory function (43).

RNA-binding motif protein 15 (RBM15) does not have a catalytic function and belongs to the split end (Spen) protein family. RBM15 can interact with METTL3 in a WTAP-dependent manner, and facilitates recruitment of the WTAP-METTL3 complex to specific RNA-binding sites, allowing m6A methylation of adjacent consensus motifs, as does its paralog, RBM15B (44, 45). Vir-like m6A methyltransferase associated (VIRMA, originally known as KIAA1429), localizes to nuclear speckles in humans, and modulates region-selective methylation modification by recruiting the catalytic core complex, METTL3-METTL14-WTAP, to specific RNA sites (46, 47). Alternatively, VIRMA associates with cleavage polyadenylation specificity factor subunit 5 (CPSF5) as well as cleavage polyadenylation specificity factor subunit 6 (CPSF6), in an m6A-dependent manner (47). In concert with WTAP, zinc finger Cys-Cys-Cys-His (CCCH)-type containing 13 (ZC3H13) retains the MTC in nuclear speckles, possibly due to its low-complexity domains, which target proteins to sub-cellular organelles enriched for RNA processing enzymes and pre-mRNA splicing factors, leading to enhanced m6A modification (48). Cbl protooncogene-like 1 (CBLL1, also called Hakai), a ring-finger type E3 ubiquitin ligase, has an essential role in maintaining the stability of MACOM (49).

In addition, there are a number of newly-discovered methyltransferases that warrant research attention. For example, CCHC-type containing 4 (ZCCHC4), which has a conserved “DPPF” catalytic motif, was identified as a novel ribosomal RNA (rRNA)-adenosine-methyltransferase, with a critical role in catalyzing m6A modification of 28S rRNA and that mediates ribosome subunit distribution and global translation (50, 51). Methyltransferase-like 5 (METTL5) participates in 18S rRNA methylation and can form a heterodimeric complex together with TRMT112, which functions as an allosteric adaptor to enhance METTL5 stabilization (52, 53). Furthermore, methyltransferase-like 16 (METTL16) is a class-I methyltransferase considered to be a unique m6A regulatory factor, distinct from the METTL3/METTL14 complex (54). METTL16 interacts directly with eIF3a, eIF3b, and rRNAs in the cytosol, to promote translation of mRNAs and deposits m6A into mRNA targets in the nucleus (55, 56). The N-terminal methyltransferase domain and two C-terminus vertebrate-conserved regions of METTL16 are RNA-binding domains used to combine with its targets (57). It binds multiple RNAs, such as pre-mRNAs, ncRNAs and lncRNAs. METTL16 serves as a m6A methyltransferase that recognizes the conserved sequence UACAGAGAA, in MAT2A mRNA and U6 snRNA substrates (58); however, the interaction between METTL16 and its substrate lncRNA MALAT1, occurs through specific recognition and binding of triple helix RNA, and whether MALAT1 is methylated by METTL16 remains to be determined (59).

The above-mentioned “writers”, particularly those investigated in only a small number of studies, require further investigation.

### 2.2 m6A “erasers”

m6A demethylases, also referred to as m6A “erasers”, can reverse m6A methylation and include: fat mass and obesity-associated protein (FTO), alkB homologue 5 (ALKBH5) and alkB homologue 3 (ALKBH3); these three proteins belong to the dioxygenase ALKB family, which have reserved α-ketoglutarate and iron(II)-dependent oxygenase domains, and can reduce m6A modification levels in RNA (60).

The first protein to be shown to harbor m6A demethylation capacity was FTO, which is located in the cytoplasm and has an important role in the regulation of adipogenesis (61). Under demethylase FTO catalysis, m6A can be sequentially oxidized to N6-hydroxymethyladenosine (hm6A) and then N6-formyladenosine (f6A), with subsequently conversion of f6A to adenosine (A), thereby regulating levels of m6A (62).

ALKBH5 was the second m6A demethylase to be discovered and is distributed in the nucleoplasm, where it is responsible for regulating mRNA export from the nucleus to the cytoplasm, as well as affecting mRNA metabolic processes, including splicing and stability (63, 64). ALKBH3, is located in both the cytoplasm and nucleus, and was recently identified as a novel m6A demethylase with a preference for demethylation of transfer RNA (tRNA), rather than mRNA or rRNA (65). Importantly, ALKBH3-demethylated tRNA prominently facilitated translation efficiency of protein (66).

### 2.3 m6A “readers”

In addition to “writers” and “erasers”, m6A modification requires another essential group of molecules, RNA binding proteins (RBPs), also termed “readers” (7). Although these proteins do not directly change the level of methylation like methyltransferases and demethylases, they can confer the destinies of RNAs by recognizing and preferentially combining the methylation sites of RNA (34).

The YT521-B homology (YTH) domain family members, YTHDF1/2/3 and YTHDC1/2, have conserved m6A-binding domains and selectively bind to RNA with m6A modification at RRACH consensus sequences, with numerous downstream effects (67, 68). YTHDF1, a translation promoter, can evoke m6A-modified mRNA translation through interaction with the translation complex, which comprises eukaryotic translation initiation factor 3 (eIF3), eIF4G, and poly (A) binding protein (PABP), among other molecules (22, 37). YTHDF2, as the first discovered and the most widely studied m6A “reader”, can recruit the deadenylase complex CCR4-NOT by direct interaction with the superfamily homology (SH) domain of CNOT1, thereby resulting in degradation of the transcripts (69, 70). with a synergistic role to those of YTHDF1 and YTHDF2, coordinating with YTHDF1 to trigger m6A-labelled mRNA translation, or cooperating with YTHDF2 to accelerate m6A-containing mRNA degradation (23, 71). Distinct from the dominant cytosol localization of other YTH domain family members, YTHDC1 is preferentially distributed in the nucleus, and contributes to pre-RNA splicing by recruiting serine- and arginine-rich splicing factor 3 (SRSF3) and antagonizing SRSF10 to promote exon inclusion (72). In addition, YTHDC1 facilitates RNA nuclear export via interacting with nuclear RNA export factor 1 (NXF1) and three prime repair exonuclease (TREX) (73, 74). YTHDC2 improves target RNA translation efficiency and elongation, as well as reducing their stability and abundance, by interacting with 5′-3′ exoribonuclease 1 (XRN1) (75, 76). The m6A “reader”, heterogeneous nuclear ribonucleoprotein (HNRNP) A2/B1 (HNRNPA2B1), localizes to the nucleus, and can not only recognize and combine with m6A-marked mRNA to regulate alternative splicing events, but also participates in facilitating miRNA maturation through binding to m6A-labelled primary miRNA transcripts and recruiting the microprocessor complex protein, DiGeorge syndrome critical region 8 (DGCR8) (19, 77, 78). Additionally, “m6A switch”, a special mechanism, has been proven to be related to both HNRNPC and HNRNPG, which can optionally bind to this structure switch, instead of directly binding to m6A sites, thereby influencing m6A-labelled transcript abundance and alternative splicing (17, 79). The three insulin-like factor-2 mRNA-binding protein (IGF2BP) paralogs, IGF2BP1/2/3, are a class of m6A “readers” located in the cytosol, and can stabilize m6A-modified target transcripts and enhance target mRNA translation efficiency by recognizing the consensus GG(m6A)C motif (26). A recent study showed that IGF2BP proteins may also be able to read the structural changes mediated by the “m6A-switch” (80).

In addition, eIF3 also serves as an m6A “reader”, located in both the nucleus and cytosol, that binds m6A sites in the 5′-UTR region of mRNAs and stimulates transcripts translation in a cap-independent manner (30). The cytosolic protein fragile X mental retardation protein (FMRP), can modulate m6A-dependent mRNA nuclear export and stability (81, 82). Further, proline rich coiled-coil 2A (PRRC2A) is a newly-discovered m6A modification “reader”, that localizes in both the nucleus and cytosol, whose GRE domain combines with a consensus GGACU motif in the Olig2 mRNA coding sequence, thereby post-transcriptionally modulating target mRNA stability in an m6A-dependent manner (83, 84). Most recently, the RBP NF-κB activating protein (NKAP) was shown to facilitate target mRNA splicing and maturation in an m6A-dependent manner (85). Further, leucine-rich pentatricopeptide repeat-containing (LRPPRC) was identified as a novel “reader” that can enhance target PD-L1 mRNA stabilization in an m6A-dependent manner (86). The specific mechanisms involved in m6A reader functions warrant further study and there remain many undiscovered “readers” to be explored.

### 2.4 Others

In addition to the deposition of m6A methylation on RNA transcripts by m6A MTCs, recent studies have found that external factors, such as histone modifications, RBPs, transcription factors, and RNA polymerase II (RNAPII), are also involved in regulating m6A deposition. It was demonstrated that m6A deposition on RNAs in mammals was linked to the histone mark, H3K36me3, which interacts directly with METTL14 to recruit the m6A MTC to bind adjacent to RNAPII, thereby promoting m6A deposition on newly formed RNA (87). Another report has found that histone H3K36me2 distributed at the 3’-end of the genes is significantly correlated with m6A deposition in the Arabidopsis genome (88). Histone H1 modification is also proposed to mediate m6A modification of nascent RNAs. Genes transcribed slowly contain high levels of histone H1, which leads to reduced RNAPII recruitment at transcription start site-proximal regions, thus facilitating co-transcriptional m6A deposition (89). Further, RNAPII transcriptional dynamics are associated with m6A deposition; slow RNAPII transcription elongation and low rates of RNAPII pausing result in elevated m6A deposition on mRNAs and decreased translation efficiency (90). In addition, the RBP, TARBP2, promotes m6A deposition on transcripts by recruiting the MTC, leading to intron retention and nuclear decay (91). In addition to mechanisms involved in activation of m6A deposition, a recent study has found that exon junction complexes (EJCs) act as m6A “suppressors”, to prevent m6A deposition in average-length internal exons by packaging proximal RNA, thereby regulating global m6A specificity (92). Although we have gained new insights into the processes that guide and suppress m6A deposition, so far, the mechanisms underlying m6A specific enrichment in certain transcriptome regions remain unclear.

## 3 Angiogenesis

### 3.1 Sprouting angiogenesis

The critically traditional tumor angiogenesis, also termed as sprouting angiogenesis, was first proposed by Professor Judah Folkman in 1971 suggesting that tumor growth was closely associated with angiogenesis (93). Since then, there have been numerous studies worldwide focused on confirming the theory of Folkman, which have led to considerable achievements based on targeting tumor angiogenesis (94, 95). Sprouting angiogenesis, existing in physiological and pathological processes, is the development of new blood vessels from pre-existing vascular networks involved in the survival and development of tumor through the supply of oxygen, nutrients as well as removal of metabolic waste, and plays a pivotal role in tumor invasion and metastasis (1, 96). Solid tumor survival and growth rely on sufficient blood supply, and when tumors reach > 2 mm in diameter, diffusion alone cannot meet their need to obtain oxygen and nutrients, so that neovascularization becomes necessary to meet the requirements of tumor tissues, which will otherwise undergo necrosis as a result of ischemia and hypoxia (1, 97). Hypoxia in cells leads to increased expression of hypoxia-inducible factor (HIF) (98), which induces upregulation of vascular endothelial (VE) growth factor (VEGF), angiopoietin (Ang), and other angiogenic molecules (99, 100). Other drivers of tumor angiogenesis include genetic mutations, inflammatory responses, and mechanical stress (101). The normal process of angiogenesis is strictly controlled by pro- and anti-angiogenic regulatory factors, to maintain a relatively dynamic homeostasis (102), disruption of which within the tumor microenvironment activates the so-called “angiogenic switch”, thus promoting tumor angiogenesis (103). Among them, pro-angiogenic factors include VEGF, platelet-derived growth factor (PDGF), epidermal growth factor (EGF), tumor necrosis factor-α (TNF-α), interleukin-8 (IL-8) etc., while anti-angiogenic factors comprise angiostatin, endostatin, thrombospondin-1 (TSP-1), tissue inhibitors of metalloproteinases (TIMPs) and so on (101, 104). Unlike normal blood vessels, tumor vasculature is abnormally variable in shape and structure, resulting in dense and disordered vascular networks (105). This is because tumor endothelial cells are not organized according to traditional grading arrangements. The characteristics of tumor blood vessels are also reflected in the permeability of curled and dilated abnormal blood vessels and the efficiency of tissue perfusion, resulting in irregular blood flow (106). Therefore, abnormalities in tumor blood vessels affect both the delivery of oxygen and nutrients to the tumor, and create a hypoxic, acidic, and inflammatory tumor microenvironment, which activates tumor angiogenesis by promoting secretion of proangiogenic factors and further inducing malignant tumor development (107, 108).

### 3.2 Vasculogenic mimicry

More than 20 years after the concept of classical tumor angiogenesis was proposed, the distinct process of vasculogenic mimicry (VM) was first defined by Maniotis et al. in human melanoma (109). VM relies on pluripotent embryonic stem cells, highly invasive tumor cells and the extra-cellular matrix in aggressive primary and metastatic tumors rather than depending on vascular ECs; however, it can also play an important role in supplying malignant tumors with sufficient blood, thus promoting tumor survival, invasion and metastasis (110). Furthermore, VM is significantly linked with tumor grade and poor prognosis in patients with aggressively malignant cancers (111, 112), consisting of gastric cancer (113), colorectal cancer (114), hepatocellular carcinoma (115), glioblastoma (116), lung cancer (117), breast cancer (118), ovarian cancer (119), among others. This explains one of the reasons why some current clinical treatments against tumor angiogenesis have not achieved satisfactory efficacy (120, 121). Epithelial-mesenchymal transition (EMT) and cancer stem cells (CSCs) are consider significant factors contributing to the relatively complex process of VM in tumors (122). Molecules essential for development of VM include: VE-cadherin, phosphatidyl inositol 3-kinase (PI3K), erythropoietin-producing hepatocellular receptor A2 (EphA2), matrix metalloproteinases (MMPs), and VE growth factor receptor (VEGFR1) (122–124). Mounting evidence has pointed out that hypoxia is also inseparable from VM formation in multiple types of solid tumors (125, 126). In addition, vascular co-option and glomeruloid angiogenesis function in tumor angiogenesis initiation. Vascular co-option refers to the process in which tumor cells attach themselves to host capillaries to obtain a blood supply providing oxygen and nutrients for tumor growth and development, without the formation of new blood vessels, and mainly occurs in organs with an extremely high degree of vascularization, such as the brain, liver, and lungs (127). Glomeruloid angiogenesis has also been reported in melanoma, breast cancer, meningioma, and GBM (128–131). However, there is relatively little research into tumor angiogenesis and malignant processes in this context, and further in-depth analysis of the mechanisms involved is needed.

## 4 Regulation of tumor angiogenesis by m6A

**Figure 2:**
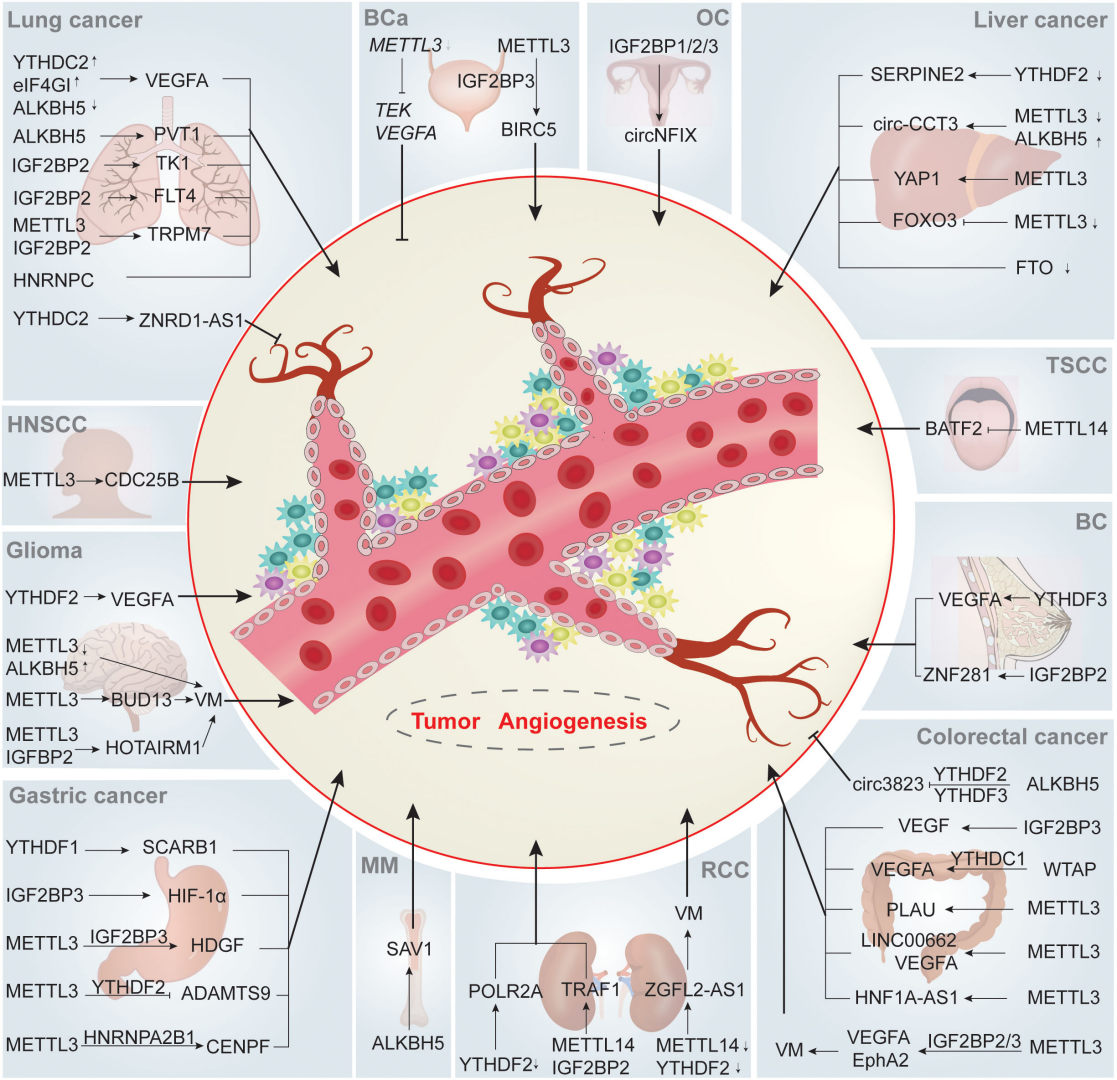
m6A regulate tumor angiogenesis. m6A writers, erasers and readers participate in tumor angiogenesis through regulating angiogenesis-related targeted proteins or pathways in an array of direct or indirect ways, ultimately influencing the tumorigenesis and development of various human tumors. ↑ and ↓ indicate upregulation and downregulation of m6A regulators, respectively. Abbreviations: BC, breast cancer; OC, ovarian cancer; HNSCC, head and neck squamous cell carcinoma; TSCC, tongue squamous cell carcinoma; BCa, bladder cancer; MM, multiple myeloma; RCC, renal cell carcinoma.

There is accumulating evidence that m6A modification regulates tumor angiogenesis in various ways, and the relationship between them is of great significance for tumor proliferation, invasion and metastasis (**Figure 2** **;** **Table 2**)

**Table 2:** m6A methylation in tumor angiogenesis.

m6A enzyme	Cancer type	Target gene	Mechanism	Result	Refs
METTL3	Colorectal cancer	VEGFA、EphA2	METTL3 mediates m6A modification of EphA2 and VEGFA to regulate the PI3K/AKT/mTOR and MEK/ERK1/2 signaling pathways, respectively.	promotes the VM formation of CRC	(128)
	Colorectal cancer	PLAU mRNA	METTL3 catalyzes m6A modification of PLAU mRNA and modulates its expression	promotes the angiogenesis of CRC	(131)
	Colorectal cancer	LINC00662、VEGFA mRNA	METTL3 regulates the stability and expression of LINC00662 and VEGFA.	promotes the angiogenesis of CRC	(132)
	Colorectal cancer	LncRNA HNF1A-AS1	METTL3 mediates HNF1A-AS1 m6A modification and affects its RNA stability.	promotes the angiogenesis of CRC	(133)
	Lung cancer	TRPM7 mRNA	METTL3 enhances m6A levels and expression of TRPM7 mRNA, and enhances the binding of IGF2BP2 and TRPM7.	promotes the angiogenesis and malignant progression of NSCLC	(151)
	Gastric cancer	HDGF mRNA	METTL3 stimulates m6A modification of HDGF mRNA and promotes HDGF nuclear export.	promotes the angiogenesis and liver metastasis of GC	(136)
	Gastric cancer	ADAMTS9 mRNA	METTL3 inhibits ADAMTS9 mRNA expression in a YTHDF2-dependent manner.	accelerates the angiogenesis and carcinogenesis of GC	(138)
	Gastric cancer	CENPF mRNA	METTL3 mediates high m6A levels in CENPF mRNA and METTL3-methylated CENPF activates the MAPK signaling pathway by facilitating FAK nuclear export.	promotes the angiogenesis of GC	(139)
	Liver cancer	YAP1 mRNA	METTL3 affects YAP1 mRNA translation efficiency in an m6A-dependent manner.	stimulates vasculogenic mimicry (VM) formation of Hepatocellular carcinoma	(141)
	Liver cancer	FOXO3 mRNA	METTL3 mediates the m6A modification of FOXO3 mRNA and improves its stability.	promotes the angiogenesis of HCC	(142)
	Liver cancer	circ-CCT3	Knockdown of METTL3 decreases m6A levels in circ-CCT3 and upregulates circ-CCT3 expression, activating the circ-CCT3/miR-378a-3p/FLT1 axis.	suppresses the angiogenesis and malignant progression of HCC	(144)
	Glioma	HOTAIRM1	METTL3-dependent m6A modification stabilizes HOTAIRM1 in glioma cells.	promotes VM formation of glioma	(147)
	Glioma	–	–	attenuates of VM formation of glioblastoma	(148)
	Glioma	BUD13	METTL3 mediates BUD13 modification and enhances its stability and expression. The BUD13/CDK12/MBNL1 axis ultimately stimulates VM in GBM.	promotes of VM formation of glioma	(149)
	Head and neck squamous cell carcinoma	CDC25B mRNA	METTL3 promotes CDC25B expression in an m6A-dependent manner.	promotes the angiogenesis and malignant progression of HNSCC	(153)
	Bladder cancer	TEK、VEGFA	Ablation of METTL3 in bladder cancer stem cells suppresses TEK and VEGFA expression.	promotes angiogenesis of bladder carcinoma	(154)
	Bladder cancer	BIRC5 mRNA	BIRC5 mRNA undergoes m6A modification by METTL3, and BIRC5 upregulates VEGF expression in bladder cells when exposed to PM2.5.	facilitates angiogenesis of bladder carcinoma	(155)
METTL14	Renal cell carcinoma	LncRNA IGFL2-AS1	Inhibition of METTL14 expression in the METTL3/METTL14 complex reduces m6A levels in IGFL2-AS1 and upregulates of AR expression in response to chronic pazopanib treatment.	inhibits VM formation in pazopanib resistance of metastatic clear cell renal cell carcinoma	(152)
	Renal cell carcinoma	TRAF1 mRNA	*TRAF1* mRNA is modified with m6A by METTL14 and overexpression of m6A-modified *TRAF1* mRNA activates AKT/mTOR/HIF1α/VEGFA signaling in sunitinib-resistant cell lines.	facilitates angiogenesis and promotes sunitinib resistance of renal cell carcinoma	(156)
	Tongue squamous cell carcinoma	BATF2 mRNA	METTL14 suppresses BATF2 mRNA expression through m6A modification. Moreover, upregulated BATF2 reduces VEGFA expression.	promotes the angiogenesis of TSCC	(158)
WTAP	Colorectal cancer	VEGFA mRNA	WTAP regulates VEGFA mRNA expression in an m6A/YTHDC1-dependent manner, thereby activating the MAPK signaling pathway.	promotes the angiogenesis of CRC	(154)
FTO	Intrahepatic cholangiocarcinoma	–	–	inhibits the angiogenesis of ICC	(160)
ALKBH5	Liver cancer	circ-CCT3	ALKBH5 cooperates with the methyltransferase, METTL3, to mediate m6A modification of circ-CCT3, thereby promoting FLT1 expression through the circ-CCT3/miR-378a-3p axis.	promotes the angiogenesis and malignant progression of HCC	(145)
	Glioma	–	–	promotes of VM formation of glioblastoma	(148)
	Lung cancer	lncRNA PVT1	Upregulated ALKBH5 slightly enhances stability and expression of the lncRNA, PVT1, and m6A-modified PVT1 mediates VEGFA expression.	promotes the angiogenesis of lung cancer	(163)
	Lung cancer	VEGFA mRNA	Loss of ALKBH5 expression effectively increases the m6A methylation and translation efficiency of VEGFA mRNA.	inhibits the angiogenesis of lung cancer	(164)
	Colorectal cancer	Circ3823	Together with YTHDF3, ALKBH5 facilities the rate of circ3823 degradation.	attenuates the angiogenesis of CRC	(166)
	Multiple myeloma	SAV1 mRNA	ALKBH5 induces m6A-demethylation of SAV1 mRNA and increases SAV1 levels.	promotes the angiogenesis of MM	(169)
YTHDC1	Colorectal cancer	VEGFA mRNA	YTHDC1 recognizes m6A sites in WTAP-mediated VEGFA mRNA, ultimately activating MAPK signaling in CRC cell lines.	promotes the angiogenesis of CRC	(154)
YTHDC2	Lung cancer	VEGFA mRNA	YTHDC2 promotes VEGFA mRNA translation in an m6A-dependent manner.	promotes the angiogenesis of lung cancer	(160)
	Lung cancer	lncRNA ZNRD1-AS1	YTHDC2 levels are significantly positively correlated with those of lncRNA ZNRD1-AS1 and regulate its stability to activate the ZNRD1-AS/miR-942/TNS1 axis and increase TNS1 levels.	suppresses the angiogenesis of lung cancer	(170)
YTHDF1	Gastric cancer	SCARB1 mRNA	YTHDF1 recognizes m6A modification sites in the 3′-UTR of *SCARB1* to facilitate its translation into SRBI through the HIF-1α/H19/YTHDF1/SCARB1 axis.	promotes the angiogenesis and malignant phenotype of GC	(175)
YTHDF2	Gastric cancer	ADAMTS9 mRNA	YTHDF2 targets ADAMTS9 mRNA and promotes its degradation in a METTL3-dependent manner.	accelerates the angiogenesis of GC	(138)
	Renal cell carcinoma	LncRNA IGFL2-AS1	Release of the YTHDF2 binding protein can stabilize METTL3/METTL14-demethylated IGFL2-AS1 in pazopanib-resistant cells.	inhibits VM formation in pazopanib resistance of metastatic clear cell renal cell carcinoma	(152)
	Renal cell carcinoma	circPOLR2A	circPOLR2A facilitates UBE3C-induced ubiquitination and degradation of PEBP1 and inhibits its expression, thus activating the ERK signaling pathway; however, YTHDF2 plays an inhibitory role in circPOLR2A expression.	suppresses the angiogenesis and malignant progression of clear cell renal cell carcinoma	(180)
	Colorectal cancer	circ3823	YTHDF2 levels are positively correlated with those of YTHDF3 and ALKBH5 and may cooperate with them to promote the rate of circ3823 degradation.	may attenuate the angiogenesis of CRC	(166)
	Liver cancer	SERPINE2 mRNA	YTHDF2 affects the expression and distribution of SERPINE2 mRNA.	suppresses the angiogenesis and malignant phenotype of HCC	(176)
	Glioma	VEGFA mRNA	YTHDF2 depletion increases the rate of VEGFA decay.	promotes of angiogenesis of glioma	(179)
YTHDF3	Breast cancer	VEGFA mRNA	YTHDF3 contributes to the translation and expression of VEGFA mRNA in an m6A-dependent manner.	promotes breast cancer brain metastasis.	(181)
	Colorectal cancer	circ3823	YTHDF3 cooperates with the demethylase, ALKBH5, to promote the rate of circ3823 degradation, stimulating the circ3823/miR-30c-5p/TCF7 axis.	attenuates the angiogenesis of CRC	(166)
IGF2BPs	Ovarian cancer	circNFIX	IGF2B1/2/3 may promote ovarian cancer angiogenesis through mediating m6A modification of circNFIX.	may promotes the angiogenesis of ovarian cancer	[188、189]
IGFBP2	Glioma	HOTAIRM1	HOTAIRM1 can positively regulate IGFBP2 expression, and IGFBP2 binds with HOTAIRM1 via METTL3-mediated m6A binding-domains and increases its stability and expression in glioma tissues and cells.	promotes vasculogenic mimicry formation of glioma	(147)
IGF2BP2	Lung cancer	TRPM7 mRNA	IGF2BP2 is positively regulated by the lncRNA, DGUOK-AS1, and functions as a bridge to provide contact between DGUOK-AS1 and TRPM7 mRNA. The METTL3/IGF2BP2 axis enhances TRPM7 mRNA stability.	promotes the angiogenesis and malignant progression of non-small cell lung cancer	(150)
	Lung cancer	FLT4 mRNA	IGF2BP2 mediates m6A modification of FLT4, improving its stability and expression. In addition, FLT4 activates the PI3K-Akt signaling pathway.	promotes the angiogenesis of lung adenocarcinoma	(183)
	Lung cancer	TK1 mRNA	The miR-320b/HNF4G axis regulates IGF2BP2 expression. Upregulated IGF2BP2 enhances TK1 mRNA stability and expression.	promotes the angiogenesis of lung cancer	(195)
	Renal cell carcinoma	TRAF1 mRNA	IGF2BP2 enhances METTL14-mediated TRAF1 mRNA stability and improves the expression of TRAF1. m6A-mediated TRAF1 activates AKT/mTOR/HIF1α/VEGFA signaling pathway in sunitinib-resistant cell lines.	facilitates angiogenesis and promotes sunitinib resistance of renal cell carcinoma	(156)
	Colorectal cancer	EphA2	IGF2BP2 participates in PI3K/AKT/mTOR signaling pathway activation by recognizing and binding to METTL3-methylated EphA2.	promotes the VM of CRC	(160)
	Breast cancer	ZNF281 mRNA	LncSNHG5 upregulates transcription and secretion of CCL2 and CCL5, and stimulates the P38 MAPK signaling pathway through the IGF2BP2/ZNF281 axis.	promotes the angiogenesis and malignant progression of BC	(192)
IGF2BP3	Gastric cancer	HDGF mRNA	IGF2BP3 enhances HDGF mRNA stability in an m6A-dependent manner.	promotes the angiogenesis and liver metastasis of GC	(136)
	Gastric cancer	HIF-1α mRNA	Under hypoxic conditions, IGF2BP3 binds to m6A modification sites in *HIF-1α* mRNA and modulates its expression.	promotes the angiogenesis of stomach cancer	(193)
	Colorectal cancer	VEGFA mRNA	IGF2BP3 is involved in recognition of and binding to METTL3-methylated VEGFA, activating the ERK1/2 pathway.	promotes the VM of CRC	(160)
	Colorectal cancer	VEGF mRNA	IGF2BP3 activates the stability and expression of VEGF mRNA.	facilitates angiogenesis of colon cancer	(182)
	Bladder cancer	BIRC5 mRNA	IGF2BP3 improves METTL3-modified BIRC5 mRNA stability, to upregulate VEGF expression in bladder cells on exposure to PM2.5.	facilitates angiogenesis of bladder carcinoma	(155)
HNRNPA2B1	Gastric cancer	CENPF mRNA	METTL3-mediated CENPF combines with HNRNPA2B1 to increase its stability. Moreover, CENPF promotes FAK nuclear export to activate MAPK signaling.	promotes the angiogenesis of GC	(139)
HNRNPC	Lung cancer	–	–	promotes the angiogenesis of NSCLC cells	(196)

### 4.1 Regulation of tumor angiogenesis by m6A writers

#### 4.1.1 METTL3

Previous publications have well documented that vascular endothelial growth factor A (VEGFA) plays a pivotal role in the formation of new blood vessels (132). Colorectal cancer (CRC) is among the most common types of cancer and presents significant challenges in terms of morbidity and mortality (133). In CRC, EphA2 and VEGFA mRNA are transcripts downstream of the “writer”, METTL3, and undergo METTL3-mediated m6A modification via different IGF2BP-dependent mechanisms, resulting in VM formation (134). Urokinase plasminogen activator (uPA, PLAU) mRNA, a classical molecule of the plasminogen activation system, contributes to various cancer processes, including tumor proliferation, invasion, metastasis, and angiogenesis (135, 136). Yu et al. demonstrated that METTL3 could facilitate angiogenesis through directly targeting and specifically catalyzing the m6A binding sites in the 3′-UTR coding region of PLAU mRNA and modulating its expression (137). m6A modification not only also functions on mRNAs, but also acts on some lncRNAs to regulate tumor angiogenesis. For example, m6A enzyme METTL3 was confirmed to modify targets―LINC00662 and VEGFA to stabilize them and positively regulate their expression levels, thus promoting angiogenesis in CRC, which was demonstrated by the levels of CD31, CD34 and VEGF (138). In addition, METTL3 mediated upregulation of lncRNA HNF1A-AS1 through influencing its stability in CRC cells, and HNF1A-AS1 overexpression can clearly promoted angiogenesis, while its deficiency has the opposite effect (139). however, the cited research was focused on exploring the mechanism underlying HNF1A-AS1 involvement in CRC cell cycle progression, while the role of HNF1A-AS1 in angiogenesis in the context of CRC has not been thoroughly elucidated.

Gastric cancer (GC) is one of the most prevalent malignant cancers worldwide associated with high morbidity and mortality (140). Hepatoma-derived growth factor (HDGF) expression is associated with aggressive biological characteristics, including cancer cell proliferation, apoptosis, angiogenesis, and metastasis (141). In GC, METTL3 stimulates m6A modification of HDGF mRNA, while the m6A “reader”, IGF2BP3 (also known as IMP3 or KOC), can stabilize HDGF mRNA through direct recognition and binding with its m6A sites. HDGF can be translocated from the nucleus to the cytoplasm and contributes to facilitating angiogenesis, ultimately promoting tumor cell growth and liver metastasis (142). ADAMTS9 mRNA, which is an independent prognostic factor in patients with GC (143), is downstream of METTL3, and ADAMTS9 mRNA m6A levels were clearly decreased after METTL3 knockdown, while the opposite effect occurred on METTL3 overexpression. Further, ADAMTS9 mRNA is inhibited by METTL3 via a YTHDF2-dependent pathway, thus accelerating GC angiogenesis (144). In addition, Xu et al. revealed that METTL3-mediated m6A modification of centromere protein F (CENPF) mRNA can enhance GC angiogenesis both *in vitro* and *in vivo*; METTL3-methylated CENPF mRNA had increased stability after combining with the m6A “reader”, HNRNPA2B1. Moreover, CENPF mRNA can promote focal adhesion kinase (FAK) nuclear export, leading to MAP kinase (MAPK) signaling pathway activation, and thereby inducing angiogenesis in GC (145).

Liver cancer is a highly aggressive tumor in humans that contributes to significant cancer-related mortality and morbidity in the world (133). Activation of yes-associated protein (YAP) mRNA has been demonstrated to contribute to tumor cell proliferation, angiogenesis and invasion in various types of tumors (146). In HCC, Qiao et al. found that YAP1 mRNA stimulates VM in an m6A-dependent manner, both *in vitro* and *in vivo*, where METTL3 mediates YAP1 m6A modification, affecting its translation efficiency (147). Lin et al. found that, under hypoxia, decreased METTL3 leads to increased expression of angiogenic markers, including FGF, PDGF-B, STAT3, and VEGFA. The mechanism underlying angiogenesis in liver cancer involves METTL3-mediated m6A modification of FOXO3 mRNA to increase its stability through a YTHDF1-dependent mechanism, which ultimately enhances sorafenib resistance of HCC (148). Further, an oncogenic role of circ−CCT3 was validated in HCC cells (149). Qian et al. conducted tube formation assays, demonstrating human umbilical vein EC (HUVEC) angiogenesis inhibition after circ-CCT3 knockdown. Mechanistically, circ-CCT3 functions as a sponge for miR-378a-3p, thereby regulating the expression of FLT1, which acts as a cell-surface receptor for VEGFA, participating in angiogenesis (150), and has a critical role in promoting HCC progression. Knockdown of the m6A methyltransferase, METTL3, caused elevated circ-CCT3 expression and a decrease of its m6A levels (151).

Glioma is the most common malignant primary brain tumor (152). Wu et al. found that HOXA transcript antisense RNA myeloid-specific 1 (HOTAIRM1), as an oncogene, is highly expressed in both glioma tissues and cell lines (the higher grade, the more expression). METTL3-dependent m6A modification imparts HOTAIRM1 stability in glioma cells, and m6A-modified HOTAIRM1 transcript plays a vital role in the promotion of VM formation (153). Tao et al. analyzed data from The Cancer Genome Atlas (TCGA) database and found that patients with relatively high METTL3 expression had prolonged overall survival (OS). Further, METTL3 downregulation, resulting in decreased RNA m6A methylation, strengthened VM formation in GBM (154). In another study, METTL3 was found to be important in enhancing the stability and expression of target BUD13 mRNA. Methylated BUD13 was bound by the downstream target, CDK12, to regulate its stability and expression, thereby promoting MBNL1 phosphorylation by CDK12, and ultimately stimulating VM in GBM (155).

Lung cancer is a significant cause of cancer-associated deaths, imposing a substantial health burden (133). Feng et al. showed that METTL3 increases m6A levels and expression of transient receptor potential melastatin 7 (TRPM7) mRNA, and enhances the binding of IGF2BP2 and TRPM7, ultimately facilitating angiogenesis in non-small cell lung cancer (NSCLC) (156). Renal cell carcinoma (RCC) is a common urological malignant cancer and represents a leading threat to healthcare (157). Cheng et al. demonstrated that METTL3/METTL14 complex-mediated lncRNA IGFL2-AS1 m6A methylation results in VM formation in pazopanib resistant metastatic clear cell RCC (ccRCC) (158). METTL3-mediated m6A modification of CDC25B is upregulated in head and neck squamous cell carcinoma (HNSCC), and promotes HNSCC malignant characteristics, including cell proliferation, migration, and invasion, as well as angiogenesis (159). In bladder cancer (BCa), Wang and coworkers found that METTL3 ablation in BCa CSCs suppressed tumor angiogenesis by regulating TEK and VEGFA (160). Liu et al. proposed that PM2.5 exposure may induce m6A methylation levels in BCa. Following exposure to PM2.5, aberrantly upregulated METTL3 modifies the 3′-UTR of BIRC5 with m6A, and IGF2BP3 combines with BIRC5 to improve its stability, ultimately accelerating angiogenesis in BCa in VEGF-dependent manner (161).

Overall, these studies illustrate the close connection between METTL3 and tumor angiogenesis, and demonstrate that METTL3 has potential as a target for cancer diagnosis and treatment. However, the differential expression of METTL3 in various cancers and its dual regulatory effect on tumor angiogenesis suggest that we should pay attention to the development and application of METTL3 activators and inhibitors.

#### 4.1.2 METTL14

In RCC, tumor necrosis factor receptor-associated factor 1 (TRAF1) mRNA is modified with m6A by METTL14, and its stability is enhanced after binding with IGF2BP2. m6A-modified TRAF1 mRNA overexpression obviously activated AKT/mTOR/HIF-1α/VEGFA signaling pathway to facilitate angiogenesis, thus promoting sunitinib resistance in RCC (162). Cheng et al. reported that IGFL2-AS1 is an m6A-modified lncRNA in pazopanib sensitive ccRCC cells. Chronic pazopanib treatment reduced the m6A level of IGFL2-AS1 and increased its expression through inhibiting METTL14 expression in METTL3/METTL14 complex. IGFL2-AS1 binds the 5′-UTR of androgen receptor (AR) mRNA and promoted AR expression, ultimately leading to VM formation and pazopanib resistance in ccRCC (158). In tongue squamous cell carcinoma (TSCC), Wen et al. found that low protein expression of basic leucine zipper ATF-like transcription factor 2 (BATF2), also termed as a suppressor of AP-1 regulated by interferon (SARI) (163), was correlated with poor patient OS duration. The m6A methylase METTL14 mediates m6A modification of BATF2 mRNA to suppress its expression. BATF2 mRNA can constrain TSCC cell angiogenesis through downregulating VEGFA (164). Further, METTL14 and the demethylase, ALKBH5, can control the expression of one another, block the demethylase activity of the m6A reader, YTHDF3, and regulate m6A modification levels of angiogenesis-associated transcripts, resulting in tumor angiogenesis and malignant processes (165). As an important component of an m6A MTC, the mechanisms underlying the effects of METTL14 on angiogenesis in other cancers warrant further investigation.

#### 4.1.3 WTAP

The methyltransferase WTAP acts as an oncogene and tumor promoter, and is significantly elevated in patients with CRC. WTAP has a pivotal role in regulation of VEGFA mRNA expression in an m6A/YTHDC1-dependent manner, which subsequently activates the MAPK signaling pathway to influence angiogenesis in CRC cell lines (166).

Whether other m6A methyltransferases are also involved in tumor angiogenesis, or affect tumor angiogenesis through other regulatory mechanisms, remains to be explored.

### 4.2 Regulation of tumor angiogenesis by m6A erasers

#### 4.2.1 FTO

Through Kaplan-Meier analysis, Rong et al. found that low levels of FTO expression were associated with inferior OS of patients with intrahepatic cholangiocarcinoma (ICC). Unlike previous studies, which reported that FTO is highly expressed in various cancers (167), levels of FTO were reported to be downregulated in clinical ICC samples and cell lines. It was detected that CD34, an indicator representing angiogenesis ability and micro-vessel density (MVD), was highly expressed in low FTO expression samples (168). However, the specific functional mechanism of m6A modification involved requires further exploration. Although the roles of FTO in tumor occurrence and development, self-renewal of CSCs, immunity, and metabolism have been extensively explored, its function in tumor angiogenesis is still poorly understood.

#### 4.2.2 ALKBH5

In lung cancer, Shen et al. demonstrated that upregulated ALKBH5 slightly enhances the stability and expression of lncRNA plasmacytoma variant translocation 1 (lncRNA PVT1). Overexpression of PVT1 partially recuperates the lung cancer angiogenesis constrained by ALKBH5 knockdown through mediating VEGFA expression (169). In contrast, Zhang et al. found that loss of ALKBH5 promotes lung cancer angiogenesis in an m6A-dependent manner. Levels of ALKBH5 are negatively correlated with those of VEGFA in patients with lung cancer, and effectively decrease the m6A methylation and translation efficiency of VEGFA mRNA, but had no effect on its mRNA levels (170). Additionally, Jin et al. demonstrated that expression of the angiogenesis-related protein, YAP, is negatively associated with that of ALKBH5, and that ALKBH5 inhibits the malignant progression of NSCLC cells by reducing YTHDFs-mediated YAP expression and suppressing miR-107/LATS2-mediated YAP activity in a HuR-dependent manner (171). In colorectal cancer, Guo and his colleagues found that ALKBH5 was significantly decreased and promoted the degradation rate of circ3823 along with YTHDF2 and YTHDF3 (172). Mechanistically, circ3823, which is closely associated with inferior patient prognosis, inhibits expression of miR-30c-5p through functioning as a competing endogenous RNA, as widely reported in various cancers (173, 174), subsequently promoting the expression of TCF7 and regulating the downstream targets, MYC and CCND1. Tube junction formation of HUVEC observation indicated that the circ3823/miR-30c-5p/TCF7 axis might facilitate the ability of angiogenesis in CRC (172). In HCC, the ALKBH5 demethylase cooperates with the methyltransferase, METTL3, in regulating circ-CCT3 m6A levels and expression, contributing to HCC growth, migration, and angiogenesis (151). In GBM, Tao et al. conducted Kaplan-Meier survival analysis demonstrating that ALKBH5 overexpression was associated with reduced patient OS duration. Further, ALKBH5 upregulation enhances VM by reducing target RNA m6A methylation (154). Yu et al. found that angiogenesis was restrained in multiple myeloma (MM) after knockdown of the demethylase, ALKBH5, both *in vivo* in and vitro, as demonstrated by VEGF secretion ability. Mechanistically, ALKBH5 promotes MM angiogenesis by inducing m6A-demethylation of SAV1 mRNA (175). In summary, ALKBH5 has a dual regulatory effect on tumors angiogenesis.

### 4.3 Regulation of tumor angiogenesis by m6A readers

#### 4.3.1 YTHDC1/2

In CRC, m6A “reader” YTHDC1 recognized specific m6A sites on VEGFA mRNA to activate WTAP/YTHDC1/VEGFA/MAPK axis, thus promoting the CRC angiogenesis (166). In lung cancer, Zhang et al. demonstrated that the m6A regulators, YTHDC2 and eIF4G, trigger angiogenesis by promoting VEGFA mRNA translation (170). Moreover, m6A “reader” YTHDC2, as a tumor suppressor that is significantly positively correlated with lncRNA zinc ribbon domain-containing 1-antisense 1 (lncRNA ZNRD1-AS1) and regulates its stability. The ZNRD1-AS1/miR-942/TNS1 axis participates in lung cancer angiogenesis regulation via YTHDC2 (176). In the early stage, the team has demonstrated that YTHDC2 was downregulated in lung cancer cells and contributed to cell proliferation, migration and the EMT process (76). In GC, YTHDC2 recognized at 5′-UTR of m6A-modified YAP mRNA, resulting in the enhancement of YAP translation efficiency, thus promoting the malignant progression of GC (177).

#### 4.3.2 YTHDF1/2/3

It is established that HIF-1α mRNA is highly expressed in cancers (178) and that HIF-1α mRNA overexpression supports cancer progression through various mechanisms, including tumor cells proliferation, invasive, metastasis, as well as angiogenesis (179, 180). In GC, Bai et al. found that the HIF-1α/H19/YTHDF1/scavenger receptor class B member 1 (SCARB1) axis is involved in angiogenesis and malignant phenotype. Mechanistically, m6A “reader” YTHDF1, functions as a bridge between lncRNA H19 and SCARB1 mRNA, and can recognize m6A modification sites on 3′-UTR of SCARB1 to facilitate its translation into scavenger receptor class B type I (SR-BI), ultimately promoting GC cells angiogenesis (181). Further, the m6A “reader”, YTHDF2, targets ADAMTS9 mRNA by recognizing its m6A motifs and promotes its degradation in a METTL3-dependent manner. Suppression of ADAMTS9 expression facilitates angiogenesis and carcinogenesis in GC (144). In CRC, YTHDF3 cooperates with the demethylase, ALKBH5, to promote the rate of circ3823 degradation, contributing to CRC growth, metastasis and angiogenesis through the circ3823/miR-30c-5p/TCF7 axis. Although it has been confirmed that interaction between YTHDF2 and YTHDF3 can promote target mRNA degradation, whether YTHDF2 participates in circ3823 degradation along with YTHDF3 and ALKBH5 requires verification (172). Additionally, YTHDF2 in HCC specimens was downregulated in contrast to normal liver histiocytes. YTHDF2 deficiency resulted in the escalation of vessel abnormity and angiogenesis of HUVECs, while overexpression of YTHDF2 reduced vessel density and permeability (182). Moreover, serpin family E member 2 (SERPINE2) mRNA, which induces angiogenesis in breast cancer and oral squamous cell carcinoma (183, 184), is upregulated after YTHDF2 knockdown and responsible for the disruption of normal vascularization (182). In glioma, Dixit et al. reported that YTHDF2 was upregulated in mesenchymal GBM and its depletion increased the VEGFA transcript decay rate in GBM stem cells in an m6A-dependent manner, thus affecting tumor angiogenesis (185). In ccRCC, circPOLR2A acts as an oncogene closely correlated with malignancy, and YTHDF2 suppresses circPOLR2A expression in an m6A-dependent manner, where the circPOLR2A/PEBP1 axis positively affects angiogenesis in ccRCC (186). Moreover, Cheng et al. demonstrated that IGFL2-AS1 was demethylated by the METTL3/METTL14 complex and stabilized by release of the binding-protein YTHDF2 in pazopanib-resistant cells. Stabilized and highly expressed IGFL2-AS1 favored AR mRNA translation and expression, leading to VM formation and development in ccRCC (158). Chang and colleagues found that the YTHDF3 upregulates its own protein expression through automatic regulation and then binds to m6A-enriched VEGFA mRNA to increase VEGFA expression and angiogenesis in brain metastases of breast cancer in humans (187). Furthermore, In NSCLC cells, YTHDFs have a crucial role in regulation of YAP expression; YTHDF3 can bind YAP pre-mRNA, while YTHDF1 and YTHDF2 regulate YAP mRNA expression through competitively interacting with YTHDF3. Further, YTHDF2 promotes YAP mRNA decay by recruiting the AGO2 degradation system, whereas YTHDF1 interacts with eIF3a to facilitate YAP translation (171). Taken together, YTHDFs represent promising potential diagnostic biomarkers and therapeutic targets.

#### 4.3.3 IGF2BPs

In CRC, both LINC00662 and VEGFA contribute to angiogenesis mediated through m6A by the enzyme METTL3. Interestingly, although high expression of IGF2BP1 in CRC was confirmed by analyzing data from TCGA CRC and the CPTAC protein prediction database, it has no effect on stabilization of RNAs (138). In patients with CRC, the m6A “reader” IGF2BP3 activates VEGF mRNA stability and expression by recognizing and combining with its m6A modification sites in an m6A-dependent manner. Moreover, interfering with IGF2BP3 represses angiogenesis in colon cancer, which was confirmed by HUVECs assay (188). Additionally, IGF2BP3 and IGF2BP2 are involved in recognition and binding to METTL3-methylated EphA2 and VEGFA, respectively, thereby enhancing the stability of these target genes and preventing their degradation. Subsequently, EphA2 and VEGFA activate the PI3K/AKT/mTOR and ERK1/2 signaling pathways, respectively, stimulating VM formation (166). AKT signaling pathway can be provoked to participate cancer angiogenesis. In RCC, In RCC, METTL14 activity modifies TRAF1 mRNA by m6A modification, and its stability was enhanced after binding with IGF2BP2. Further, m6A-modified TRAF1 mRNA overexpression obviously activated AKT/mTOR/HIF-1α/VEGFA signaling pathway to facilitate angiogenesis, thus promoting sunitinib resistance in RCC (162). In lung cancer, Shen and colleagues demonstrated that IGF2BP2 permeates ECs in the microenvironmental via lung adenocarcinoma (LUAD) cell-derived exosomes, subsequently mediating the m6A modification of FLT4 to improve its stability and expression. Then, FLT4 activates the PI3K-Akt signaling pathway, eventually promoting angiogenesis in LUAD cells (189).

Besides AKT effector, several factors are also involved in the regulation of angiogenic process, including p38 MAPK (190), FAK (191), signal transducer and activator of transcription 1 (STAT1) (192)and Rho GTPases (193). In ovarian cancer (OC), Ye et al. found that OC-derived exosomal circRNA nuclear factor IX (circNFIX) regulates the Janus-activated kinase (JAK)/STAT1 pathway via the miR-518a-3p/TRIM44 axis, thereby promoting tumor angiogenesis (194). The team further validated that IGF2BP1/2/3 recognizes m6A modification sites in circNFIX, leading to increased circNFIX expression in OC cells (195). Therefore, IGF2BP1/2/3 may promote OC angiogenesis by regulating circNFIX; however, the specific mechanism involved requires further in-depth investigation. Moreover, Zeng et al. showed that the LncSNHG5/ZNF281 axis upregulates transcription and secretion of CCL2 and CCL5, thereby activating P38 MAPK signaling in HUVECs, ultimately stimulating angiogenesis and vascular permeability in a VEGF-independent manner (196). Mechanistically, LncSNHG5 is highly increased in breast cancer-associated fibroblasts, which are an important subset of stromal fibroblasts in the tumor microenvironment (197). Enhanced LncSNHG5 has a key role in regulating angiogenesis and vascular leakiness by mediating recruitment of IGF2BP2 to augment ZNF281 mRNA stabilization, thus inducing lung premetastatic niche formation, which is reported to be essential for malignant tumor progression (198).

VEGF is key for tumor angiogenesis and is upregulated by HIF and other oncogenic factors. In GC, Jiang et al. demonstrated that IGF2BP3 binds to m6A sites on HIF-1α mRNA in stomach cancer (SC) cells to positively modulate HIF-1α mRNA expression in an m6A-dependent manner and promote angiogenesis in SC through the IGF2BP3/HIF-1α pathway under hypoxic conditions (199). Further, the HIF-1α/VEGF axis can contribute to mediation of angiogenesis and GC cell malignant growth in a hypoxic microenvironment (200). Inhibition of VEGF secretion by IGF2BP3 deficiency can be alleviated by HIF-1α mRNA overexpression (199). However, further exploration is needed to determine whether a methylase or demethylase regulates the IGF2BP3/HIF-1α/VEGF axis. In BCa, Liu et al. found that IGF2BP3 combines with METTL3-modified BIRC5 mRNA to improve its stability, ultimately accelerating angiogenesis in BCa exposed to PM2.5 in a VEGF-dependent manner (161). Wang and his colleagues verified that m6A “reader” IGF2BP3 recognized and bound m6A sites on METTL3-mediated HDGF mRNA to stabilize it and the subsequent GC angiogenesis and malignant progress (142).

Ma et al. found that hepatocyte nuclear factor 4 gamma (HNF4G) is a target of miR-320b, where HNF4G expression is reduced on miR-320b overexpression. HNF4G upregulates the m6A reader, IGF2BP2, by combining with its promoter region, and upregulation of IGF2BP2 enhances the thymidine kinase 1 (TK1) stability and expression, thus facilitating angiogenesis in lung cancer (201). IIGF2BP2 functions as a bridge between the lncRNA, deoxyguanosine kinase antisense RNA 1 (DGUOK-AS1) and TRPM7 mRNA, where TRPM7 is positively regulated by DGUOK-AS1 in NSCLC; hence, the DGUOK-AS1/IGF2BP2/TRPM7 axis promotes angiogenesis in NSCLC (156). In glioma, IGFBP2 is closely associated with METTL3-mediated HOTAIRM1 via its m6A binding-domains and positively regulated by HOTAIRM1, thus promoting VM (153). Collectively, IGF2BPs appear to promote tumor angiogenesis, and targeted inhibition of IGF2BP expression may suppress tumor angiogenesis to some extent.

#### 4.3.4 HNRNPs

In gastric cancer, m6A reader HNRNPA2B1 directly bound m6A sites of METTL3-methylated CENPF mRNA and promoted the mRNA stability, ultimately GC angiogenesis and metastasis through activating the FAK/MAPK axis (145). Additionally, Gu et al. reported that HNRNPC predicted poor prognosis in patients with NSCLC, and was associated with NSCLC angiogenesis by Gene Ontology and Gene Set Enrichment Analysis, although the exact mechanism involved remains unclear (202).

#### 4.3.5 Others

**Figure 3:**
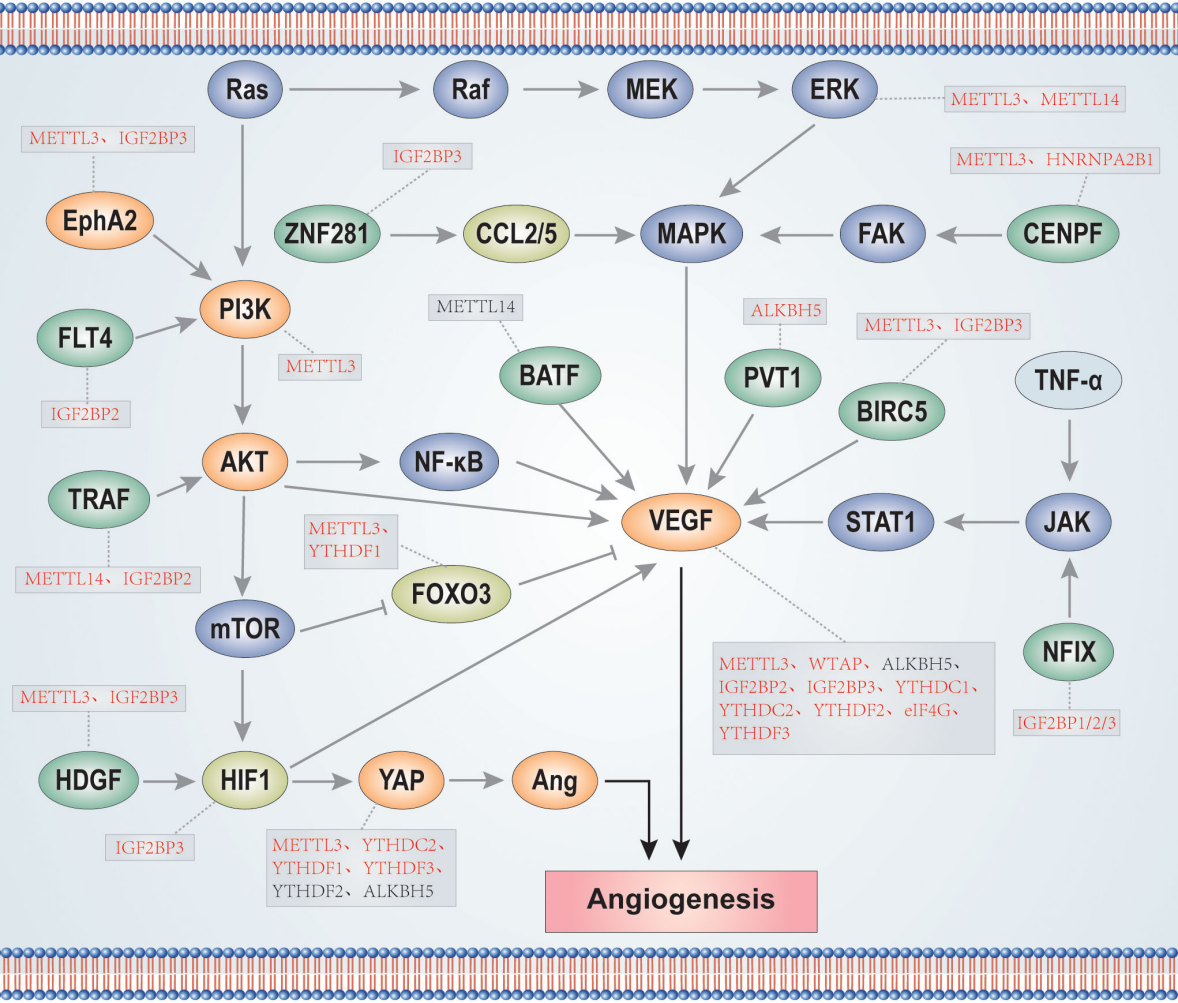
m6A enzymes participate in the regulation of signaling pathways and proteins related to tumor angiogenesis. The red font indicates that m6A enzymes positively regulate proteins, while the black font indicates that m6A enzymes negatively regulate proteins.

He et al. analyzed 24 main m6A RNA methylation regulators from the TCGA breast cancer dataset, divided them into two subsets according to the height of RNA methylation modification-RNA methylation 1 (RM1) and RM2, and identified that angiogenesis was significantly comfortable in RM1 (203). Further, Li et al. showed that m6A modification in glioma stem cells is mainly distributed around sites of neovascularization in hypoxic environments. Further, the overall interaction between angiogenesis-related genes(ARGs) and m6A regulators(MAGs) is significantly correlated in low-grade gliomas (LGGs) (204). These findings provide potential new research directions for investigation of the relationship between m6A and angiogenesis, including deeper studies to generate more valuable and practical guidance that can inform cancer treatment. In summary, VEGF is the main factor involved in promotion of tumor angiogenesis, while other factors, such as Ang, can coordinate with VEGF in this context. Enzymes involved in m6A methylation modification and regulation play crucial roles in tumor angiogenesis, not only by direct regulation of VEGF expression, but also through mediating angiogenesis-related signaling pathways, such as PI3K/AKT, MAPK, and JAK/STATA, to affect VEGF expression (**Figure 3**). Nevertheless, numerous m6A regulators have not been investigated in the context of tumor angiogenesis and study of mechanisms involving such m6A regulators may be a fruitful research direction. Further, exploration of the interactions between the three types of m6A enzymes during tumor angiogenesis is of interest.

## 5 The clinical significance of m6A methylation in tumor angiogenesis

m6A modification has become a hot topic of current research, attracting various researchers who have focused on in-depth investigation into its roles in pathogenesis and malignant phenotypes. As described above, numerous studies have shown that m6A-regulated tumor angiogenesis and VM have crucial roles in tumor occurrence and development. In this section, we discuss the potential clinical significance of targeting m6A-regulated tumor angiogenesis.

### 5.1 Anti-angiogenesis therapy

Currently, the clinical therapeutic strategy of anti-angiogenesis is among the most commonly used treatment schemes to control tumor proliferation and distant metastasis. Since the concept of targeted angiogenesis was proposed, the US Food and Drug Administration has approved numerous anti-angiogenic drugs (93), which can be divided into three main categories: first, monoclonal antibodies, including bevacizumab and ramucirumab; second, fusion proteins, of which Ziv-aflibercept is an example; and third, VEGFR-targeting small molecules, including sorafenib, sunitinib, pazopanib, lenvatinib, and tivozanib, among others (205). For example, sunitinib is a tyrosine kinase inhibitor that shows potent anti-angiogenic activity, and was recommended as a first-line targeted drug for patients with recurrent and unresectable RCC (206–208). However, the majority of patients with RCC eventually develop drug resistance and malignant tumor progression, resulting in inability of sunitinib to effectively prolong their survival (209, 210). Angiogenesis switch is among the mechanisms elucidated as involved in the development of resistance to sunitinib (211), and is also associated with high expression of TRAF1 mRNA; increased TRAF1 expression contributes to activation of downstream anti-apoptotic and anti-angiogenic pathways in sunitinib-resistant cells, leading to drug resistance in patients with RCC (162). In combination with standard first-line chemotherapy drugs (including cisplatin and carboplatin), the monoclonal antibody, bevacizumab, can significantly prolong progression-free survival (PFS) and OS in patients with advanced NSCLC, as demonstrated by a meta-analysis of randomized studies (212). other drugs are currently under investigation and are expected to be used in clinical anti-angiogenesis treatment of tumors in the future. For example, using xenograft and chorioallantoic membrane angiogenesis models, as well as detection of CD31 via IHC assay, Wei et al. demonstrated that tumor angiogenesis is inhibited after treatment with verteporfin, which downregulates angiopoietin-2 (Ang2) by suppressing YAP activity (213). The correlation between Ang2 and YAP has also been clarified in previous studies, where Ang2 was reported as an important biomarker of vasculogenic events (214), and YAP also functions to promote angiogenesis through regulating angiogenic germination and remodeling of HUVECs (215). Verteporfin can also repress VM by downregulating MMP2, VE-cadherin, and a-SMA expression (216). In addition, RSK and TTK were identified as novel modulators of angiogenesis and potential targets for anti- angiogenic therapy (217). In accordance with the findings of these studies, resistance of tumors to anti-angiogenic drugs is a complex process involving multiple genes, factors, mechanisms, and the tumor microenvironment. Changing the expression of certain key transcripts in angiogenesis and activating or inhibiting specific signaling pathways may enhance the sensitivity of tumors to treatment. Hence, identification of new targets for anti-angiogenesis treatment and combining them with other drugs could provide new avenues for exploration.

### 5.2 m6A-targeted therapy

Tumor anti-angiogenesis therapy has not achieved optimal therapeutic effects, due to the development of resistance over time, raising concerns regarding whether the combination of m6A and anti-angiogenic drugs will contribute to improved antitumor therapy. Some recent studies have discussed mechanisms involving m6A-regulated tumor angiogenesis or VM in drug treatment and resistance, and suggested that targeting m6A-regulated tumor angiogenesis also has therapeutic potential. VEGFR-targeted treatment is a common method of inhibiting tumor angiogenesis. The IGFL2-AS1/AR signaling axis is clinically associated with VM formation, and is strongly increased during pazopanib resistance of metastatic ccRCC. Mechanistically, the lncRNA, IGFL2-AS1, interacts with the 5’UTR of AR mRNA to regulate the activity of the upstream open reading frame and enhance translation of AR mRNA, which was demethylated by METTL3/METTL14, during pazopanib resistance (158).Chen et al. manifested that a novel pharmaceutical intervention strategy for the treatment of patients with sunitinib might be targeting TRAF1 mRNA and its pathways in the near future. Mechanistically, METTL14-mediated m6A modification of *TRAF1* enhances its stability in an IGF2BP2-dependent manner, upregulating TRAF1 expression in sunitinib resistant cells. In addition, TRAF1 overexpression significantly stimulates AKT/mTOR/HIF-1α/VEGFA signaling, while silencing TRAF1 increases sunitinib-induced anti-apoptotic and anti-angiogenic effects (162). Further, Lin et al. found that the methyltransferase, METTL3, is dramatically down-regulated in human sorafenib-resistant HCC. METTL3 depletion decreases the stability of its downstream target, FOXO3, in a YTHDF1-dependent manner and reduces FOXO3 expression levels, ultimately promoting sorafenib resistance (148). hence, targeting METTL3 expression may enhance treatment response to sorafenib. In many types of cancer, tumor angiogenesis is also accompanied by increased YAP mRNA expression levels and activity. The m6A demethylase, ALKBH5, decreases YTHDF-mediated YAP expression to suppress tumor growth and metastasis in NSCLC cells (171). Further, IGF2BP2 recognizes m6A sites on YAP mRNA and facilitates its translation efficiency in CRC cells. The IGF2BP2/YAP/ErbB2 axis promotes CRC cells proliferation, invasion, and migration and represses CRC cell apoptosis (218). Therefore, targeting m6A enzymes which mediate YAP expression and activity could be a promising therapeutic strategy. This suggests the new idea that m6A can increase the sensitivity of anti-angiogenic drugs through targeted regulation; therefore, combination therapy using m6A inhibitors or activators together with angiogenesis inhibitors, is expected to improve anti-tumor efficacy.

Nevertheless, there has been relatively little research to date on the mechanism underlying targeting of m6A combined with anti-angiogenesis approaches, and further in-depth research is needed to determine the clinical value of such methods, in terms of anti-tumor therapy and survival improvements for patients with cancer. Overall, m6A regulatory factors are of great significance in tumor prognosis and diagnosis. Some m6A-related molecules are currently in clinical trials, and m6A is expected to become a drug target for clinical anti-cancer treatment in the future (**Table 3**).

**Table 3:** Clinical trials of m6A regulators related to cancer. (Data from ClinicalTrials.gov).

Study title	Condition(identifier)	Status	Interventions	Phase	Study design	N
Oral Administration of STC-15 in Subjects With Advanced Malignancies	Advanced Solid Tumor or Cancer (NCT05584111)	Recruiting	Drug: STC-15	I	I; NR; SA; OL	66
Peptide Vaccination in Treating Patients With Esophageal Cancer	Esophageal Cancer (NCT00682227)	Unknown	Biological: TTK, LY6K, and IMP-3 peptides	I	I; SGA; OL	10
Imiquimod Treatment of CIN Lesions	Cervical Intraepithelial Neoplasia (NCT02329171)	Terminated	Drug: Imiquimod; Procedure: LLETZ	III	I; R; PA; OL	9
Histocompatibility Leukocyte Antigen (HLA)-A*2402 Restricted Peptide Vaccine Therapy in Patients With Esophageal Cancer	Esophageal Cancer (NCT00681330)	Completed	Biological: URC10, TTK, KOC1	I、II	I; SGA; OL	14
Safety Study of Cancer Specific Epitope Peptides Cocktail for Cervical, GI, and Lung Tumors (peptidevac)	Metastatic Tumors (NCT00676949)	Completed	Biological: 5 peptide vaccines of KOC1, TTK, CO16, DEPDC1, MPHOSPH1	I	I; SGA; OL	18
Histocompatibility Leukocyte Antigen (HLA)-A*2402 Restricted Peptide Vaccine Therapy in Patients With Non-Small Cell Lung Cancer	Non Small Cell Lung Cancer (NCT00674258)	Unknown	Biological: URLC10, TTK and KOC1	I、II	I; SGA; OL	14
Histocompatibility Leukocyte Antigen (HLA)-A*2402 Restricted Peptide Vaccine Therapy in Patients With Gastric Cancer	Gastric Cancer (NCT00681577)	Completed	Biological: URLC10, KOC1, VEGFR1 and VEGFR2	I、II	I; SGA; OL	14
Combination of Chemoradiation Therapy and Epitope Peptide Vaccine Therapy in Treating Patients With Esophageal Cancer	Esophageal Cancer (NCT00632333)	Unknown	Biological: URLC10, TTK, KOC1, VEGFR1, VEGFR2, cisplatin, fluorouracil	I	I; SGA; OL	9
Peptide Vaccination in Treating Patients With Esophageal Cancer (STF-II)	Esophageal Cancer (NCT01267578)	Unknown	Biological: vaccination	II	I; SGA; OL	60

I, interventional; NR, non-randomized; SA, sequential assignment; OL, open label; SGA, single group assignment; R, randomized; PA, parallel assignment.

## 6 Conclusions

The connections between tumors and angiogenesis have been explored for more than five decades, since they were first proposed by Professor Folkman. The rapid development of high-throughput sequencing technologies, popularization of bioinformatics, and emergence of highly specific antibodies, have led to verification that m6A methylation is involved in various tumor malignant processes, including proliferation, invasion, metastasis, and immune escape, as well as angiogenesis. At present, there is an increasing research focus on the relationship between m6A and tumor angiogenesis, and m6A modification has been found to directly or indirectly regulate tumor cell angiogenesis. In different types of tumor, m6A modification influences biological behaviors through affecting the stability and expression of target mRNAs and activating or repressing angiogenesis-related signaling pathways, to regulate tumor angiogenesis. Further, m6A modification also occurs in ncRNAs, including lncRNAs, miRNAs, and circRNAs, and m6A-modified ncRNAs can impact their downstream signaling axes, thereby regulating tumor angiogenesis in an m6A-dependent manner. There is a growing body of evidence suggesting that m6A modification regulates tumor angiogenesis and malignant phenotypes through an extremely complex interaction network, resulting in influences on the occurrence, development, treatment, and prognosis of cancer. Further study of the potential mechanisms underlying the relationships between m6A methylation and tumor angiogenesis will improve understanding and provide novel possibilities for tumor diagnostic methods and therapeutic strategies in the near future. At present, the effectiveness of anti-angiogenic drugs for treating patients with cancer is suboptimal. The strategy of targeting m6A in combination with anti-angiogenic drugs or vascular mimicry inhibitors is predicted to open a promising new frontier for future tumor treatment; however, enormous research challenges remain. According to existing research, it is clear that only a proportion of m6A regulators have been investigated, and the relationships between other m6A regulators and tumor angiogenesis remains unclear. Further exploration is needed to determine whether other factors that promote or suppress m6A deposition contribute to tumor angiogenesis. Additionally, m6A regulators have a dual regulatory effect on tumor angiogenesis in different cancers; and even within the same cancer, different researchers hold opposite views. Differences among tumor microenvironments and upstream and downstream genes may be important factors affecting the expression and function of m6A regulators. Therefore, multi-center, large-scale research could provide deeper and more comprehensive understanding of mechanisms involving m6A in tumor angiogenesis, and facilitate screening and development of specific inhibitors or activators targeting m6A. Given the extensive research on the mechanisms underlying the role of m6A modification in tumor angiogenesis, the development of specific m6A-targeted drugs will be of great significance for achieving personalized and precise treatment and, combined with other approaches, such drugs may improve malignant tumor therapy sensitivity, reduce drug resistance and side effects, and achieve superior therapeutic effects. Although there have been numerous reports indicating that various m6A regulators have potential diagnostic, prognostic, and therapeutic value in the context of anti-tumor angiogenesis, research on the regulation of tumor angiogenesis by m6A remains in its infancy. The development of specific m6A-targeted activators or inhibitors suitable for clinical application and the translation of scientific research into clinical practice will require considerable further efforts.
